# Characteristics of long stay home care clients’ acute care use who live with frailty in Alberta, Canada: A retrospective cohort study

**DOI:** 10.1371/journal.pone.0351298

**Published:** 2026-06-10

**Authors:** Jananee Rasiah, Andrea Gruneir, Jeffrey Poss, Jayna Holroyd-Leduc, Greta G. Cummings

**Affiliations:** 1 College of Health Sciences, Faculty of Nursing, University of Alberta, 3-141 Edmonton Clinic Health Academy (ECHA), Edmonton, Alberta, Canada; 2 College of Health Sciences, Faculty of Medicine and Dentistry, University of Alberta, 6-10 University Terrace, University of Alberta, Edmonton, Alberta, Canada; 3 School of Public Health and Health Systems, Faculty of Applied Health Sciences, University of Waterloo, Lyle Hallman Institute (LHN) 3727 200, University Avenue West, Waterloo, Ontario, Canada; 4 Cumming School of Medicine, University of Calgary, Foothills Medical Centre, Calgary, Alberta, Canada; Tokyo Metropolitan Institute of Geriatrics and Gerontology, JAPAN

## Abstract

**Objective:**

To describe the characteristics of long stay home care clients’ acute care use who live with frailty, and to compare associated factors for hospitalizations.

**Methods:**

The cohort comprised individuals 65 to 104 years old (n=10,107) who were observed from 2015-2017. Population-based administrative data were linked by individual records, descriptive statistics were reported, and logistic regression models were run.

**Results:**

Majority of clients were female (69.7%), and the largest proportion of clients were 75-84 years old. Arthritis (65.6%) and hypertension (74.5%) were most prevalent in clients who were frail. Frail clients (40.6%) had between 1-3 emergency department visits. Mean length of stay was 16.6 days for frail hospitalized clients. Being pre-frail and frail were associated factors for hospitalization.

**Conclusion:**

Further research is needed to determine the risk classification of more factors, such as social characteristics that predispose home care clients with frailty to hospitalization.

## Introduction

Older adults living with frailty are increasingly vulnerable to stressors because of deficits in multiple physiological systems that in turn affect their ability to maintain adequate homeostatic reserve functions. Community dwelling older adults living with frailty rely heavily on home care services for their complex care needs. Frailty is a dynamic process, whereby older adults’ needs change over time depending on the stressors that they are exposed to [1]. Stressors include illnesses or injuries that can lead to emergency department (ED) visits or hospitalizations and result in increased risks of adverse outcomes, such as worsening levels of frailty, falls, delirium, dependency, institutionalization, and mortality. Since frail older adults are more vulnerable to stressors, it is reasonable to consider that frail older adults are also at increased risk of developing adverse events that will lead to ED visits, hospitalization, and re-hospitalization [2,3]. In Canada, approximately 50% of patients admitted to hospital after ED visits are over the age of 65 [4]. These older adults present to EDs more acutely ill than younger patients and are thus more likely to be hospitalized [5–7]. Fall-related injuries and other self-care issues are among the most common reasons for ED visits among older adults [7].

Authors in a meta-analysis found that frailty and prefrailty in community dwelling older adults across the globe were significantly associated with increased risk of hospitalization (N=10 studies; pooled odds ratios [OR]=1.90, 95% CI 1.74–2.07, p<0.00001; pooled OR=1.26, 95% CI 1.18–1.33, p<0.00001, respectively) [8]. However, the degree of frailty appears to be a factor in hospital admissions. In an Australian study, authors found those who were most frail had lower number of hospital admissions (incidence rate ratio 0.65, 95% confidence interval [CI] 0.42–0.99) and length of stay (LOS) in hospital (in days) (incidence rate ratio 0.39, 95% CI 0.33–0.46) [9]. In comparison, residents living with mild or moderate frailty had the highest number of hospital admissions (adjusted incidence rate ratio 1.57, 95% CI 1.11–2.20) and LOS (incidence rate ratio 1.48, 95% CI 1.32–1.66) [9].

Being able to predict who is at highest risk for hospitalization among those utilizing home care would be helpful in prioritization of services. Predictive risk of hospitalization in 1-year for residents in assisted living in Alberta was measured using the Changes in Health, End-stage disease and Signs and Symptoms (CHESS), two different frailty indexes (FIs), and the Fried Frailty Phenotype, which showed no significant difference of predictive risk among these tools [2,10]. Alternatively, the CHESS was found to be a better predictor of hospitalization than two different FIs in a retrospective cohort study of home care clients (older adults) in Ontario [11]. This was an expected finding, as the CHESS was originally developed to predict mortality in institutionalized older adults, and is a valid and reliable measure of instability in health and a good predictor of hospitalization [12].

Hospitalization can itself contribute to changes in the level of frailty. Among community dwelling older adults, the chance of improvement from increased levels of frailty was reduced with multiple hospitalizations [13,14]. Hospitalizations in older adults were also strongly associated with new or increasing levels of disability or physical frailty [15]; worsening Activities of Daily Living (ADL) function [16]; and cumulative loss of ADL and Instrumental Activities of Daily Living (IADL) functioning at three months post-discharge [17]. The association between frailty and increased risk of hospitalization may be muted by other associated factors such as risk of mortality, availability of social support, or advanced care planning [2]. Muted in this case means that the true association between two variables may be reduced, invisible, or obscured because of the presence and influence of other variables or confounders, such as risk factors. Also, patterns of acute care use vary by frailty severity and care setting, which suggests that examining frailty within specific service contexts such as home care is a priority [9]. To address this priority, we conducted a population-based retrospective cohort study of long-stay home care clients in Alberta to describe ED visits and hospitalizations by frailty level and identify key factors associated with hospitalization. The objectives of this study were to describe older adults’ characteristics of ED visits and hospitalizations across frailty groups and to identify and compare older adults’ associated factors for hospitalization, with the goal of optimizing home care services.

## Methods

### Setting

Older adults (65 years and older) comprise 15% of the 4.5 million people living in Alberta [18]. Alberta Health Services (AHS) is an integrated health system that serves Albertans and has 106 acute care hospitals across the province [19]. AHS home care services are provided for clients (with valid healthcare cards) on a long-stay or short-stay basis to help them remain safe and in good health while living at home or in designated supportive living (DSL) settings [20]. DSL is a pathway in the continuum of care between home living and long-term care (LTC) [21]. In this study, our cohort comprised of clients who received publicly funded home care services in Alberta. Our cohort resided outside of DSL and were active home care recipients on a continuous basis, meaning they were long-stay home care clients living in private residences. We chose this type of cohort because they have different characteristics, care needs, risk for hospitalization, and health outcomes in comparison to those who reside in congregate care settings with regular oversight from healthcare providers.

### Data sources

Population-based administrative datasets were linked at the individual level using encrypted identifiers and fully anonymized by the analyst from the Alberta SPOR SUPPORT Unit [22] and transferred securely to the Health Research Database Repository (HRDR) at the University of Alberta before we accessed the data for analyses. We accessed the data for this study through the HRDR on March 25, 2022. Assessment data from the Resident Assessment Instrument in Home Care (RAI-HC), which is a standardized and validated clinical assessment for long-stay home care clients, is housed within the Alberta Continuing Care Information System [23]. Functional and cognitive status were determined using outcome scales in the RAI-HC with scale score cut-offs presented as two groups based on previous studies including: ADL Hierarchy Scale [24]; IADL Difficulty [24]; Cognitive Performance Scale (CPS) [25]; and Depression Rating Scale (DRS) [26]. Frailty status was determined using the 72-item frailty index, which is a derived measure from items in the RAI-HC with final cut points of robust (<0.2), pre-frail (0.2-0.3), and frail (>0.3) [11,27]. Items selected from the RAI-HC to derive the 72-item frailty index were included in S1 Appendix.

The RAI-HC assessments were linked with data from the National Ambulatory Care Reporting System (NACRS), which contains standardized reporting on all emergency department (ED) visits [28,29]; Discharge Abstract Database (DAD), which contains standardized chart abstractions on all hospitalizations [28,30]; Practitioner Claims database for physician visits [31]; and Pharmaceutical Information Network (PIN) Dispenses database for community and outpatient prescriptions. These datasets have been studied extensively for validity and are commonly used for research purposes [32–34]. This study was approved by the research ethics board at the University of Alberta (ID: Pro00094280). Informed consent was waived because population level data without identifiers were obtained.

### Cohort

**Fig 1:**
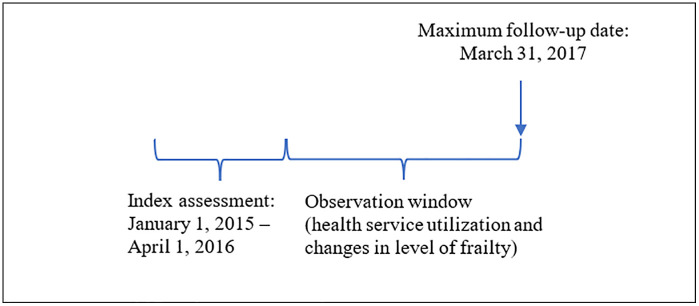
Look-back, observation, and follow-up windows.

**Fig 2:**
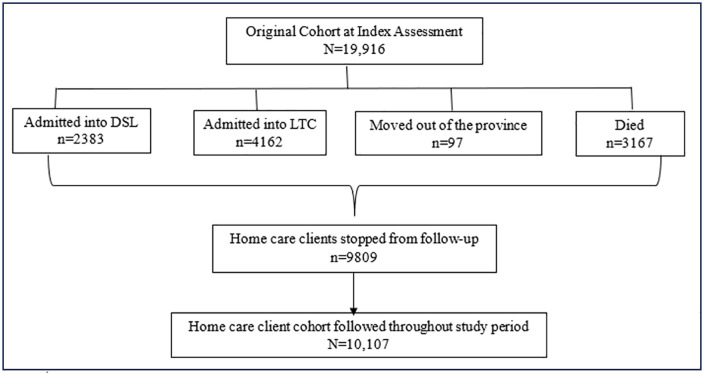
Home care client cohort followed between January 1, 2015 – March 31, 2017.

The cohort for this study was drawn from Alberta residents who were long-stay home care clients aged 65 years or older and assessed with the RAI-HC during a qualifying window. Only those who were not discharged from home care in the 1-year period starting from their index assessment were included in the analysis. The cohort was constructed in this manner to simplify loss to follow-up issues and to examine associated factors for the healthier home care population. The index assessment was a RAI-HC assessment completed between January 1, 2015 (baseline) – April 1, 2016, and if more than one assessment was completed during this period, the one closest to the baseline date was selected. Each cohort member was followed for 365 days from index assessment and those who were lost to follow-up due to discharge, death, moving out of province, or being admitted to LTC or DSL were removed from the cohort (Fig 1). Those who died or were admitted to LTC and DSL were removed from the cohort (Fig 2) to examine the characteristics of and the factors for acute care use among the ‘healthiest’ home care population. Therefore, the analytic cohort represented a select subset of long-stay home care clients who survived and remained in home care for the full follow-up period.

### Variables

The outcome variable of interest for this study was acute care use defined as ED visits and hospitalizations 1-year follow-up from index assessment. For ED visits, we counted the number of visits during the follow-up period and selected the last ED visit per client in the follow-up period for additional characterization. Due to the potential for multiple visits and the anticipated variability across clients, we chose the last ED visit to simplify the process. Three types of ED visits were of interest that were not mutually exclusive: (a) potentially preventable visits: defined as ambulatory care sensitive conditions (ACSC) [35] such as angina, asthma, chronic obstructive pulmonary disease, diabetes, epilepsy, heart failure, pulmonary edema, and hypertension were identified [36,37], (b) low acuity visits: defined by the Canadian Triage and Acuity Scale Score (CTAS) of 4-5 [38], which was used to examine the association between home care visits and ED use [39], and (c) fall-related injuries derived based on the diagnostic code. For CTAS visits, we grouped CTAS 1-3 and CTAS 4-5 visits accordingly. Some CTAS visit data were missing, as these were not coded in the dataset. We identified ED discharge disposition and categorized outcomes as admission to acute care; discharge home; or other.

Hospitalization types were defined as non-elective hospitalizations (or unplanned admissions) [40]. If more than one hospitalization occurred within a 24-hour period (based on the discharge date of one hospitalization and the admission date of a subsequent hospitalization within a 24-hour period), then these hospitalizations were counted as a single episode to account for transfers between hospitals. We counted the number of hospital episodes during the 1-year follow-up period from index assessment and kept the last hospitalization for further description. The last hospitalization was selected per client in the follow-up period for additional characterization. Due to the potential for multiple hospitalizations and the anticipated variability across clients, we chose the last hospitalization to simplify the process. We identified the length of stay (LOS), hospitalization with or without alternate level of care (ALC) days, fall-related hospitalizations, and discharge disposition from hospital (home without home care; home with home care; residential care/group or supportive living/continuing care; or other).

Demographic variables of age and sex were identified from the index assessment. Client health characteristics 1-year follow-up from index assessment were identified including: (a) most common chronic conditions and geriatric-specific concerns related to frailty, counted from the RAI-HC diagnoses list, (b) frequency of family physician visits, (c) prescription medications according to the Anatomical Therapeutic Chemical (ATC) code and anticholinergic risks, (d) functional and cognitive status using outcome scales in the RAI-HC, and (e) frailty level using the 72-item frailty index (Full FI), which is an accumulation of deficits type index (a derived measure from items in the RAI-HC) [11,27].

### Data analyses

#### Characteristics of older adults’ acute care use.

Frequency of baseline characteristics of the cohort were reported. The frequency of ED visits, type of ED visits (as described above), and discharge disposition for robust, pre-frail, and frail groups were reported. Pairwise Chi-square tests were performed to compare “robust versus pre-frail groups” and “pre-frail versus frail groups” for all ED variables that were coded as categorical. Since these pairwise Chi-square tests were exploratory, no adjustments for multiple comparisons were applied.

The frequency of hospitalization, LOS, ALC days, fall-related hospitalizations, and discharge disposition from hospital for robust, pre-frail, and frail groups were reported. The mean and standard deviation for LOS was calculated. The standardized differences were calculated and compared for “robust versus pre-frail groups” and “pre-frail versus frail groups” within a 95% CI for LOS. Standardized differences for LOS were interpreted based on Cohen’s *d*, as follows: small effect (*d*=0.2), medium effect (*d*=0.5), and large effect (*d*=0.8). Pairwise Chi-square tests were performed to compare “robust versus pre-frail groups” and “pre-frail versus frail groups” for all hospitalization variables that were coded as categorical.

#### Comparison of factors associated with hospitalization.

First, univariable (unadjusted) logistic regression models were run, where each correlate was examined separately to identify factors associated with hospitalization. ED visits were excluded from the regression model because clinically, most hospital admissions arise from ED visits, making them a strong predictor of hospitalization. Potential correlates tested in the model included, age, sex, ADL hierarchy scale, IADL difficulty scale, CPS, DRS, and frailty level. ADL hierarchy scale, IADL difficulty scale, CPS, and DRS were re-coded as dichotomous, for ease of comparison. Next, another logistic regression model was run to test the interaction between frailty levels and each correlate (new variables created) because frailty is also associated with hospitalization. Sensitivity analyses were also performed by removing each interacting new variable to test their overall influence on the model. A variable was considered as associated with higher odds of hospitalization if the estimated OR was > 1.0 and was statistically significant when the 95% CI did not include 1.0 [41]. When the OR was < 1.0, the variable was considered a protective factor. All analyses were conducted using SAS © software version 9.4 (SAS Institute Inc., Cary, NC, USA).

## Results

We identified a total of 10,107 home care clients in Alberta, who met our inclusion criteria. Cohort characteristics were presented in Table 1, of which 7,143 cohort members were robust, 2,269 were pre-frail, and 695 were frail. The largest proportion of clients were aged 75–84 years (40%), were female (70%), and belonged to the robust group. Statistically significant differences were found between the robust and pre-frail groups for all chronic conditions commonly associated with clients living with frailty. All functional and cognitive scales, prescription medication counts, and family physician visits had significant differences when compared by frailty. A statistically significant difference was found for prescription medications with anticholinergic risk (as defined on the Anticholinergic Risk Scale) between pre-frail and frail groups.

**Table 1:** Characteristics of home care clients in Alberta categorized by frailty groups as per the Full FI.

	Overall Cohort	Robust	Pre-frail	Frail		
	N=10,107	N=7,143	N=2,269	N=695		
	N (%)	N (%)	N (%)	N (%)		
*Characteristics*					robust vs pre-frail	pre-frail vs frail
*Age group*					*P value significant if ≤0.05*	
65 – 74 y	2,423 (24.0)	1,641 (23.0)	608 (26.8)	174 (25.0)	P=<0.0001	P=0.4619
75 – 84 y	3,983 (39.4)	2,801 (39.2)	908 (40.0)	274 (39.4)		
85 – 105 y	3,701 (36.6)	2,701 (37.8)	753 (33.2)	247 (35.5)		
*Sex*						
Male	3,064 (30.3)	2,196 (30.7)	673 (29.7)	195 (28.2)	P=0.3290	P=0.4165
Female	7,043 (69.7)	4,947 (69.3)	1,596 (70.3)	500 (71.9)		
*Comorbidities*						
Osteoporosis	2,168 (21.5)	1,332 (18.7)	624 (27.5)	212 (30.5)	P=<0.0001	P=0.1238
Arthritis	5,666 (56.1)	3,777 (52.9)	1,433 (63.2)	456 (65.6)	P=<0.0001	P=0.2387
Alzheimer disease	368 (3.6)	194 (2.7)	114 (5.0)	60 (8.6)	P=<0.0001	P=0.0004
Dementia	1,261 (12.5)	624 (8.7)	411 (18.1)	226 (32.5)	P=<0.0001	P=<0.0001
Hypertension	6,881 (68.1)	4,642 (65.0)	1,721 (75.9)	518 (74.5)	P=<0.0001	P=0.4801
Congestive heart failure	1,162 (11.5)	612 (8.6)	414 (18.3)	136 (19.6)	P=<0.0001	P=0.4327
Lung disease (Emphysema/COPD/asthma)	1,976 (19.6)	1,192 (16.7)	590 (26.0)	194 (27.9)	P=<0.0001	P=0.3176
Diabetes	2,786 (27.6)	1,726 (24.2)	808 (35.6)	252 (36.3)	P=<0.0001	P=0.7549
*ADL Hierarchy Scale*						
0 (independent)	8,347 (82.6)	6,541 (91.6)	1,545 (68.1)	261 (37.6)	P=<0.0001	P=<0.0001
1 or 2 (setup only)	1,183 (11.7)	480 (6.7)	517 (22.8)	186 (26.8)		
3 (extensive assistance/total dependence)	577 (5.7)	122 (1.7)	207 (9.1)	248 (35.7)		
*IADL Difficulty Scale*						
0 (no difficulty performing IADLs)	1,263 (12.5)	1,206 (16.9)	51 (2.3)	6 (0.9)	P=<0.0001	P=<0.0001
1 or 2 (some difficulty performing IADLs)	3,400 (33.6)	2,864 (40.1)	495 (21.8)	41 (5.9)		
3 (some/great difficulty performing IADLs)	5,444 (53.9)	3,073 (43.0)	1,723 (75.9)	648 (93.2)		
*Cognitive Performance Scale*						
0 (no impairment)	5,803 (57.4)	4,830 (67.6)	874 (38.5)	99 (14.2)	P=<0.0001	P=<0.0001
1 or 2 (moderate impairment)	3,839 (38.0)	2,231 (31.2)	1,240 (54.7)	368 (53.0)		
3 (moderate to severe impairment)	465 (4.6)	82 (1.2)	155 (6.8)	228 (32.8)		
*Depression Rating Scale*						
0 (no mood symptoms)	6,910 (68.4)	5,761 (80.7)	1,004 (44.3)	145 (20.9)	P=<0.0001	P=<0.0001
1 or 2 (few mood symptoms)	1,927 (19.1)	1,101 (15.4)	680 (30.0)	146 (21.0)		
3 (depression)	1,270 (12.6)	281 (3.9)	585 (25.8)	404 (58.1)		
*Unique prescription medications by ATC Code*						
0-5	613 (6.1)	530 (7.4)	86 (3.8)	45 (6.5)	P=<0.0001	P=0.0094
6-9	1,299 (12.9)	1,026 (14.4)	207 (9.1)	66 (9.5)		
10+	8,147 (81.0)	5,587 (78.2)	1,976 (87.1)	584 (84.0)		
*ARS Scale*						
0 (no risk)	9,565 (95.1)	6,827 (95.6)	2,147 (94.6)	639 (91.9)	P=0.1489	P=0.0107
1 or 2 (low risk)	381 (3.8)	243 (3.4)	91 (4.0)	47 (6.8)		
3> (high risk)	113 (1.1)	73 (1.0)	31 (1.4)	9 (1.3)		
*Family physician visits category*						
0	69 (0.7)	45 (0.6)	13 (0.6)	11 (1.6)	P=<0.0001	P=0.0123
1-14	2,226 (22.0)	1,763 (24.7)	447 (19.7)	160 (23.0)		
15-28	3,184 (31.5)	2,274 (31.8)	703 (31.0)	207 (29.8)		
29+	4,484 (44.4)	3,061 (42.9)	1,106 (48.7)	317 (45.6)		

The features of ED visits and non-elective hospitalizations were summarized in Tables 2 and 3 respectively. There was a total of 40,432 ED visits made by 7,953 cohort members. Statistically significant differences were found for potentially preventable ED visits between the pre-frail and frail groups and for CTAS visits between the robust and pre-frail groups. There was a total of 11,105 hospital visits made by 4,986 cohort members. For LOS in hospital, statistically significant differences were found between robust and pre-frail groups. Fall-related hospitalizations were statistically significant between robust and pre-frail groups.

**Table 2:** Characteristics of ED visits by groups of frailty according to the Full FI for home care clients in Alberta.

	Overall Cohort N= 10,107	Robust N=7,143	Pre-frail N=2,269	Frail N=695		
	N (%)	N (%)	N (%)	N (%)		
*Number of ED visits in follow-up period*					robust vs. pre-frail	pre-frail vs. frail
					*P value significant if ≤0.05*	
0	2,154 (21.3)	1,596 (22.3)	418 (18.4)	140 (20.1)	P=0.0002	P=0.3660
1-3	4,290 (42.4)	3,023 (42.3)	985 (43.4)	282 (40.6)		
≥4	3,663 (36.2)	2,524 (35.4)	866 (38.2)	273 (39.3)		
	Subcohort with ED visits only					
*Final ED Visit Descriptors*	Overall Subcohort N=7,953	Robust N=5,547	Pre-frail N=1,851	Frail N=555		
*Low acuity ED visits category*	N (%)	N (%)	N (%)	N (%)		
Missing CTAS* visit	138 (1.7)	101 (1.8)	32 (1.7)	5 (0.9)	P=0.0324	P=0.3804
CTAS* 1-3 (high acuity)	5,535 (69.6)	3,804 (68.6)	1,329 (71.8)	402 (72.4)		
CTAS* 4-5 (low acuity)	2,280 (28.7)	1,642 (29.6)	490 (26.5)	148 (26.7)		
*Potentially preventable ED visits*						
Not potentially preventable ED visit	7,792 (98.0)	5,441 (98.1)	1,805 (97.5)	546 (98.3)	P=0.1316	P=0.2325
Potentially preventable ED visit	161 (2.0)	106 (1.9)	46 (2.5)	9 (1.6)		
*Fall-related ED visits*						
No	6,841 (86.0)	4,750 (85.6)	1,603 (86.6)	488 (87.9)	P=0.2995	P=0.4166
Yes	1,112 (14.0)	797 (14.4)	248 (13.4)	67 (12.1)		
*Discharge disposition from the ED*						
Acute care	3,034 (38.2)	2078 (37.5)	728 (39.3)	228 (41.1)	P=0.3413	P=0.7199
Home	4,818 (60.6)	3,400 (61.3)	1,099 (59.4)	319 (57.5)		
Other	101 (1.3)	69 (1.2)	24 (1.3)	8 (1.4)		

Note: CTAS*- Canadian Triage and Acuity Scale Score

**Table 3:** Features of all non-elective hospitalization for home care clients in Alberta living with frailty categorized according to the Full FI.

	Overall Cohort N= 10,107	Robust N=7,143	Pre-frail N=2,269	Frail N=695	robust vs. pre-frail	pre-frail vs. frail
N (%)	N (%)	N (%)	N (%)	*P value significant if ≤0.05*	
*LOS category (days)*						
No hospitalizations	5,121 (50.7)	3,775 (52.9)	1,032 (45.5)	314 (45.2)	P=<.0001	P=0.0669
1-6	2,339 (23.1)	1,589 (22.3)	588 (25.9)	162 (23.3)		
7-13	1,330 (13.2)	887 (12.4)	344 (15.2)	99 (14.2)		
14+	1,317 (13.0)	892 (12.5)	305 (13.4)	120 (17.3)		
	Subcohort with Hospitalizations only					
*LOS for hospitalizations*	Overall Subcohort N= 4,986	Robust N=3,368	Pre-frail N=1,237	Frail N=381	robust vs. prefrail	pre-frail vs. frail
Mean (std) hospital LOS (days)	13.0 (21.9)	12.9 (21.6)	12.1 (17.7)	16.6 (33.4)		
Standardized difference					*d*=0.0440*	*d*=0.1786*
*Final Hospital Admission Descriptors*					robust vs. pre-frail	pre-frail vs. frail
*ALC days in hospital category (days)*	N (%)	N (%)	N (%)	N (%)	*P value significant if ≤0.05*	
0	4,230 (84.8)	2,866 (85.1)	1,052 (85.0)	312 (81.9)	P=0.9660	P=0.1389
≥1	756 (15.2)	502 (14.9)	185 (15.0)	69 (18.1)		
*Fall-related hospitalizations*						
No	4,300 (86.2)	2,869 (85.1)	1,092 (88.3)	339 (89.0)	P=0.0073	P=0.7093
Yes (≥1)	686 (13.8)	499 (14.8)	145 (11.7)	42 (11.0)		
*Discharge disposition from hospital*						
Home without home care referral/supports	1,863 (37.4)	1,244 (36.9)	478 (38.6)	141 (37.0)	P=0.2622	P=0.0964
Home with home care referral/supports	2,433 (48.8)	1,629 (48.4)	598 (48.3)	206 (54.1)		
Residential care/group/supportive living/continuing care	143 (2.9)	97 (2.9)	39 (3.2)	7 (1.8)		
Other	547 (11.0)	398 (11.8)	122 (9.9)	27 (7.1)		

*Cohen’s *d* = 0.2 (small); *d*=0.5 (medium); *d*=0.8 (large)

In Table 4, specific factors associated with increased odds of hospitalizations were summarized. Factors associated with hospitalizations were found to be statistically significant with high ORs as follows: (1) individuals with some and great difficulty performing IADLs; (2) individuals with prefrailty; and (3) individuals with frailty. Men and those with moderate or severe impairment were less likely to be hospitalized. Pre-frail individuals with moderate to severe impairment in the CPS were less likely to be hospitalized.

**Table 4:** Odds ratios for factors associated with hospital admission.

Variables	Hospital Admission
	OR [95%]
*Age Groups (ref=65-74y)* 75 – 84y	1.06 [0.95-1.17]
85 – 105y	1.12 [1.00-1.24]
*Sex (ref=Female)* Male	0.81 [0.75-0.89]
*ADL Hierarchy Scale categories (ref=independent)* Setup, extensive assistance and total dependence	0.97 [0.91-1.03]
*IADL Difficulty Scale categories (ref=no difficulty)* Some and great difficulty to perform IADLs	1.17 [1.10-1.24]
*CPS (ref=no impairment)* Moderate and severe impairment	0.88 [0.84-0.92]
*DRS (ref=no mood symptoms)* Mood symptoms and depression	0.98 [0.93-1.02]
*Full FI (ref=robust)* Pre-frail	1.09 [1.01-1.18]
Frail	1.19 [1.02-1.39]

In Table 5, new interacting variables with frailty that were created were not statistically significant, as shown in the main analysis. With the sensitivity analyses, where each interacting variable was tested to determine the effect on the model, the following interacting variables: (1) males (sex variable)*robust (frailty variable); (2) moderate to severe impairment (CPS variable)*robust (frailty variable) and (3) moderate to severe impairment (CPS variable)*pre-frail (frailty variable) were protective factors for every sensitivity analysis. Also, none of the results were substantially different when the main analysis was compared to the sensitivity analyses.

**Table 5:** Logistic regression to test interactions with the Full Frailty Index for the likelihood of hospital admission with sensitivity analyses.

Variables (*depicts the interaction between variables)	Main Analysis	Sensitivity Analysis 1	Sensitivity Analysis 2	Sensitivity Analysis 3	Sensitivity Analysis 4	Sensitivity Analysis 5	Sensitivity Analysis 6
**Hospital Admission**	**Hospital Admission**	**Hospital Admission**	**Hospital Admission**	**Hospital Admission**	**Hospital Admission**	**Hospital Admission**
**OR [95%CI]**	**OR [95%CI]**	**OR [95%CI]**	**OR [95%CI]**	**OR [95%CI]**	**OR [95%CI]**	**OR [95%CI]**
*Age Groups (ref=65-74y)* 75 – 84y*robust	1.07 [0.91-1.26]	N/A	1.07 [0.91-1.25]	1.07 [0.91-1.27]	1.06 [0.90-1.25]	1.06 [0.90-1.25]	1.06 [0.90-1.25]
75 – 84y*pre-frail	0.99 [0.67-1.46]	N/A	1.01 [0.69-1.49]	0.98 [0.67-1.44]	1.11 [0.91-1.36]	0.98 [067-1.44]	1.02 [0.70-1.50]
75 – 84y*frail	0.92 [0.44-1.94]	N/A	0.96 [0.46-2.00]	0.89 [0.43-1.86]	1.16 [0.84-1.59]	0.91 [0.44-1.89]	0.98 [0.47-2.05]
85 – 105y*robust	1.10 [0.86-1.41]	N/A	1.10 [0.86-1.41]	1.11 [0.87-1.42]	1.10 [0.86-1.41]	1.10 [0.86-1.40]	1.10 [0.86-1.40]
85 – 105y*pre-frail	1.03 [0.67-1.57]	N/A	1.04 [0.69-1.59]	1.01 [0.67-1.54]	1.14 [0.88-1.49]	1.01 [0.67-1.54]	1.05 [0.69-1.60]
85 – 105y*frail	0.96 [0.45-2.03]	N/A	0.99 [0.47-2.09]	0.92 [0.4-1.95]	1.19 [0.84-1.70]	0.94 [0.44-1.98]	1.02 [0.48-2.14]
*Sex (ref=Female)* Male*robust	0.86 [0.80-0.92]	0.86 [0.80-0.92]	N/A	0.86 [0.81-0.92]	0.86 [0.80-0.92]	0.87 [0.81-0.93]	0.85 [0.80-0.91]
Male*pre-frail	0.86 [0.60-1.24]	0.87 [0.61-1.24]	N/A	0.85 [0.59-1.21]	0.97 [0.85-1.10]	0.89 [0.62-1.27]	0.89 [0.63-1.27]
Male*frail	0.87 [0.43-1.77]	0.88 [0.43-1.78]	N/A	0.84 [0.41-1.69]	1.09 [0.85-1.39]	0.90 [0.45-1.83]	0.93 [0.46-1.88]
*ADL Hierarchy Scale categories (ref=independent)* Setup/Extensive assistance/total dependence*robust	0.94 [0.87-1.00]	0.93 [0.87-1.00]	0.94 [0.88-1.01]	N/A	0.94 [0.88-1.01]	0.93 [0.87-0.99]	0.95 [0.88-1.01]
Setup/Extensive assistance/total dependence*pre-frail	0.95 [0.65-1.38]	0.96 [0.66-1.39]	0.97 [0.67-1.40]	N/A	1.06 [0.91-1.25]	0.93 [0.64-1.35]	0.98 [0.67-1.42]
Setup/Extensive assistance/total dependence*frail	0.96 [0.46-2.02]	0.99 [0.47-2.07]	0.99 [0.47-2.07]	N/A	1.20 [0.88-1.62]	0.94 [0.45-1.95]	1.01 [0.48-2.11]
*IADL Difficulty Scale categories* *(ref=no difficulty)* Some/great difficulty to perform IADLs*robust	1.05 [0.78-1.42]	1.06 [0.78-1.44]	1.04 [0.77-1.41]	1.02 [0.75-1.38]	N/A	1.03 [0.76-1.39]	1.05 [0.78-1.43]
Some/great difficulty to perform IADLs*pre-frail	1.11 [0.80-1.53]	1.11 [0.81-1.53]	1.13 [0.82-1.55]	1.09 [0.79-1.50]	N/A	1.06 [0.77-1.45]	1.14 [0.83-1.57]
Some/great difficulty to perform IADLs*frail	1.17 [0.80-1.70]	1.17 [0.80-1.70]	1.22 [0.84-1.76]	1.16 [0.80-1.69]	N/A	1.09 [0.76-1.56]	1.24 [0.86-1.78]
*CPS (ref=no impairment)* Moderate to severe impairment*robust	0.87 [0.81-0.95]	0.87 [0.81-0.95]	0.89 [0.82-0.96]	0.87 [0.80-0.94]	0.88 [0.81-0.95]	N/A	0.88 [0.81-0.95]
Moderate to severe impairment*pre-frail	0.77 [0.54-1.11]	0.77 [0.54-1.11]	0.79 [0.55-1.14]	0.76 [0.53-1.09]	0.86 [0.75-0.99]	N/A	0.80 [0.56-1.14]
Moderate to severe impairment*frail	0.68 [0.33-1.39]	0.68 [0.33-1.39]	0.71 [0.35-1.44]	0.66 [0.32-1.34]	0.85 [0.67-1.07]	N/A	0.72 [0.36-1.47]
*DRS (ref=no mood symptoms)* Few mood symptoms/Depression*robust	0.94 [0.87-1.01]	0.94 [0.87-1.01]	0.93 [0.86-1.00]	0.96 [0.89-1.03]	0.94 [0.88-1.02]	0.94 [0.87-1.01]	N/A
Few mood symptoms/Depression*pre-frail	0.95 [0.66-1.36]	0.96 [0.67-1.38]	0.96 [0.67-1.38]	0.93 [0.65-1.33]	1.06 [0.92-1.22]	0.94 [0.65-1.34]	N/A
Few mood symptoms/Depression*frail	0.96 [0.47-1.96]	0.98 [0.48-2.01]	0.99 [0.49-2.01]	0.91 [0.45-1.84]	1.19 [0.92-1.55]	0.94 [0.46-1.90]	N/A

Sensitivity Analysis 4 excluded the IADL Difficulty Scale variable. Sensitivity Analysis 5 excluded the CPS variable. Sensitivity Analysis 6 excluded the DRS variable.

Sensitivity Analysis 1 excluded the age group variable. Sensitivity Analysis 2 excluded the Sex variable. Sensitivity Analysis 3 excluded the ADL Hierarchy Scale variable.

## Discussion

In this retrospective cohort-study, the objectives were to describe the characteristics of ED visits and hospitalizations across frailty groups and to identify and compare associated factors for hospitalizations. As expected, we found that women made up a larger proportion of home care clients than men, which is consistent with prior literature reporting a higher proportion of women among home care clients [42]. Chronic conditions that were significant in our study, such as hypertension, diabetes, and dementia were also found to be experienced commonly in this population [43]. “Being male” and “having moderate to severe cognitive impairment” were protective factors for hospitalization in our study, which contrasts with findings in other studies. For example, community dwelling older adults, who had a hospitalization within a 24-month follow-up period were included in the cohort, and those who moved to a nursing home or died were excluded [44]. For this cohort, being male was significantly associated with hospitalization; however, the authors’ decisions to limit their cohort to those who did not move to a nursing home or had died during the follow-up period [44] was similar to our criteria for cohort composition in this study. In another example, cognitive deficit (measured using the Mini-Mental Status Examination) with physical frailty (measured using the Short Performance Physical Battery Score, a proxy measure of frailty) was shown to increase the odds of community dwelling Chinese older adults to experience ED visits and hospitalizations within a 24-month follow-up period [45]. Therefore, our findings that women and home care clients with no cognitive impairment are more likely to be hospitalized, are different than findings from the two studies above. This is because our criteria for cohort composition were such that people who died or were admitted to LTC were excluded from the cohort, which means that the cohort likely did not include individuals with the most severe levels of frailty. These criteria also likely resulted in a relatively stable subset of home care clients that may have biased estimates of frailty, acute care use, and factors associated with hospitalizations. The cohort in this study was probably healthier with better survival rates at 1-year follow-up from index assessment. This is because those who needed more care beyond what is available through home care services in private residences would likely experience more severe frailty levels and may have required more acute care services. Therefore, our findings are generalizable to home care clients living with frailty who are unlikely to require LTC in the foreseeable future and/or have sufficient supports to stay in their own homes despite functional changes.

ED visits for clients receiving home care with frailty were significant, which is similar to previous findings [24,46,47]. The high degree of association may reflect unmet needs of older adults, which leads them to seek care in EDs instead of relying on their home care team. Pre-frail and frail groups of clients had higher odds for hospitalization. This is a consistent finding that frailty is an important predictor of hospitalization [8]. It is important to remember that the risk classification for hospitalization in our study was limited to a healthier home care population, as mentioned above. Therefore, examining models in home care to prevent potentially preventable or low acuity ED visits may help inform strategies to reduce ED visits and hospitalizations. For example, moving away from silos of care and working collaboratively within a comprehensive team model to monitor the health of clients within home care is a viable way forward and requires effective resource allocation [39]. Also, since the last ED visit and last hospitalization were described in detail, which represent a sample of acute care encounters, the results may not be representative of the full range of acute care encounters.

We also ran sensitivity analyses to test interactions of select factors with frailty on the likelihood of hospitalization; however, the results were not very different than the main analyses with wide confidence intervals. One of the reasons for this finding is because frailty is also a risk factor for hospitalization, as authors have indicated when comparing the prevalence of frailty in acute care settings among older adults in comparison to the prevalence in population studies [48–50]. Also, since an “a priori” measure for frailty does not exist in the RAI-HC and proxy measures in the RAI-HC (such as, the CHESS) or derived measures from the RAI-HC such as the various frailty indexes are used, comparison across studies becomes challenging. For studies that used frailty indexes derived from the RAI-HC, including the Full FI that we used in our study, the challenge is the increased degree of correlation of the items that were tested in the models (from standalone items in the other outcome scales) with those contained in the Full FI.

Overall, the decision to examine individuals eligible for home care only (i.e., cohort composition) could have affected the demographic profile and health characteristics of the resultant frailty groups and their associated factors for hospitalization. For example, although we know that home care is a publicly funded and available service to Albertans, there are inequities in home care access for those living in rural/remote areas, who are recent immigrants, and have cultural/language barriers [51–54], who could have likely been missed in our cohort.

Finally, mechanisms that underpin the association between frailty and subsequent increased risk of hospitalization require further investigation. For example, in other studies, authors found that social characteristics such as loneliness and lack of caregiver or informal care had strong associations for hospitalizations in pre-frail and frail clients [55–57]. Therefore, exploring loneliness and caregiver/informal care and risk of hospitalization in the home care population by frailty groups would be useful.

## Conclusions

In this study, individual level factors for hospitalization were reported by frailty status for a select cohort of long-stay home care clients who survived and remained in home care for one year. Further investigation of factors associated with hospitalization that predispose home care clients with frailty to hospitalization warrants further investigation to inform targeted interventions. Examining individual level deficits for pre-frail or frail clients according to the Full FI index, can inform individualized home care plans to help inform approaches aimed at reducing hospitalizations. Exploring the associations of social isolation and lack of caregiver or informal care as risk factors for hospitalization may assist to optimize home care supports.

## Supporting information

S1 Appendix RAI-HC items for Full FI* (DOCX)
