# Supplementary material for: Characteristics of long stay home care clients’ acute care use who live with frailty in Alberta, Canada: A retrospective cohort study
Source: PLoS One. 2026 Jun 10;21(6):e0351298. doi: 10.1371/journal.pone.0351298 (PMC13252772; doi:10.1371/journal.pone.0351298)
Supplement: S1 Appendix — (DOCX) [file pone.0351298.s001.docx]

**S1 Appendix.** RAI-HC items for Full FI*

| **RAI-HC item description** | **Full frailty index (72 items denoted by** ✓ **)** |
| --- | --- |
| ***Psychosocial well-being*** |  |
| Recent decline in level of participation in social activities | ✓ |
| Socially isolated during day | ✓ |
| Client indicates feelings of loneliness | ✓ |
| ***Mood*** |  |
| Makes negative statements | ✓ |
| Exhibits persistent anger | ✓ |
| Expresses unrealistic fears | ✓ |
| Repetitive health complaints | ✓ |
| Repetitive anxious complaints | ✓ |
| Sad, pained, worried facial expressions | ✓ |
| Crying, tearfulness | ✓ |
| Withdrawal from activities of interest | ✓ |
| Reduced social interactions | ✓ |
| ***Cognition*** |  |
| Impaired cognitive skills for daily decision making | ✓ |
| Short-term (5-min recall) memory problem | ✓ |
| Procedural memory problem | ✓ |
| New onset/worsening of mental function in past 7 days | ✓ |
| Change in decision making in past 90 days | ✓ |
| ***Communication*** |  |
| At least some difficulty to make self-understood | ✓ |
| At least some difficulty in understanding others | ✓ |
| Moderate/severe hearing problems | ✓ |
| Moderate/severe vision problems | ✓ |
| ***Functional Status and Activity Level*** |  |
| At least some difficulty with phone use | ✓ |
| Needs help with stairs | ✓ |
| At least some difficulty with shopping | ✓ |
| Requires at least some assistance with bathing | ✓ |
| Requires at least some assistance with personal hygiene | ✓ |
| Requires at least some assistance with dressing upper body | ✓ |
| Requires at least some assistance with dressing lower body | ✓ |
| Requires at least some assistance with locomotion | ✓ |
| Requires at least some assistance with transferring | ✓ |
| Requires at least some assistance with toilet use | ✓ |
| Requires at least some assistance with bed mobility | ✓ |
| Requires at least some assistance with eating | ✓ |
| Less than 2 hours of physical activity in past 3 days | ✓ |
| Does not leave house at all in typical week | ✓ |
| Decline in an Activity of Daily Living in last 90 days | ✓ |
| ***Incontinence*** |  |
| Some to Daily bladder incontinence | ✓ |
| Some to Daily bowel incontinence | ✓ |
| ***Disease Diagnoses*** |  |
| Hip fracture, other fractures, osteoporosis | ✓ |
| Arthritis | ✓ |
| Alzheimer disease/Dementia | ✓ |
| Head Trauma | ✓ |
| Hemiplegia | ✓ |
| Multiple sclerosis | ✓ |
| Parkinsonism | ✓ |
| Stroke or CVA | ✓ |
| Hypertension | ✓ |
| Coronary artery disease | ✓ |
| Congestive heart failure | ✓ |
| Emphysema/COPD/asthma | ✓ |
| Cancer | ✓ |
| Diabetes | ✓ |
| Renal failure | ✓ |
| Peripheral vascular disease | ✓ |
| Irregularly irregular pulse | ✓ |
| Thyroid disease | ✓ |
| ***Presence of other health conditions*** |  |
| At least one fall in last 90 days | ✓ |
| Dizziness in past 3 days | ✓ |
| Unsteady gait | ✓ |
| Chest pain | ✓ |
| Delusions | ✓ |
| Hallucinations | ✓ |
| Vomiting present last 3 days | ✓ |
| Edema | ✓ |
| Shortness of breath | ✓ |
| Pain | ✓ |
| Client reports poor self-rated health | ✓ |
| Unstable health condition | ✓ |
| ***Nutritional status and medications*** |  |
| Severe malnutrition | ✓ |
| Morbid obesity | ✓ |
| Unintended weight loss of 5% or more in past 30 days / 10% or more in past 180 days | ✓ |
| 9 or more medications | ✓ |

*Adapted from Campitelli et al. 2016
